# Yap/Taz transcriptional activity in endothelial cells promotes intramembranous ossification via the BMP pathway

**DOI:** 10.1038/srep27473

**Published:** 2016-06-07

**Authors:** Mami Uemura, Ayumi Nagasawa, Kenta Terai

**Affiliations:** 1 Laboratory of Function and Morphology, Institute of Molecular and Cellular Biosciences, The University of Tokyo, Yayoi 1-1-1 Bunkyo-ku, Tokyo, 113-0032 Japan

## Abstract

Osteogenesis is categorized into two groups based on developmental histology, intramembranous and endochondral ossification. The role of blood vessels during endochondral ossification is well known, while their role in intramembranous ossification, especially the intertissue pathway, is poorly understood. Here, we demonstrate endothelial Yap/Taz is a novel regulator of intramembranous ossification in zebrafish. Appropriate blood flow is required for Yap/Taz transcriptional activation in endothelial cells and intramembranous ossification. Additionally, Yap/Taz transcriptional activity in endothelial cells specifically promotes intramembranous ossification. BMP expression by Yap/Taz transactivation in endothelial cells is also identified as a bridging factor between blood vessels and intramembranous ossification. Furthermore, the expression of Runx2 in pre-osteoblast cells is a downstream target of Yap/Taz transcriptional activity in endothelial cells. Our results provide novel insight into the relationship between blood flow and ossification by demonstrating intertissue regulation.

Osteogenesis is anatomically categorized into intramembranous ossification or endochondral ossification^1^,^2^. Intramembranous ossification directly forms bones from mesenchyme cells, while endochondral ossification involves cartilage as a precursor. During endochondral ossification in mammalian systems, the invasion of blood vessels into the cartilage is a well-known process for converting cartilage to bone. Blood vessels in the cartilage provide osteoclast cells, which differentiate from macrophages^3^,^4^. Vascular endothelial growth factor (VEGF) secreted by chondrocytes also promotes the replacement of cartilage^5^,^6^. In Zebrafish (*Danio rerio*), the involvement of vessels in ossification is still unknown^7^.

Yes-Associated Protein (Yap) and transcriptional co-activator with PDZ-binding motif (Taz), are transcriptional factors identified as downstream participants in the Hippo pathway^8^,^9^,^10^,^11^. Yap/Taz regulates many aspects of cell behavior including proliferation, survival, and differentiation^10^,^12^. The Hippo pathway is a regulator of organ size and is activated via the phosphorylation of mammalian STE20-like protein kinases 1 and 2, as well as large tumor suppressor kinases 1 and 2 13. The Hippo pathway suppresses cell proliferation in high cell density environments by inhibiting the nuclear translocation of Yap/Taz. Recently, it was revealed that the complex of angiomotin family members (AMOT, AMOTL1 and AMOTL2) and F-actin also inhibits Yap/Taz nuclear translocation by retaining it in the cytoplasm^14^. Since Yap/Taz localization and transactivation are regulated by substrate stiffness and fluid shear stress^15^, Yap/Taz is also regarded as a mechanosensor.

The role of Yap/Taz transactivation in promoting osteogenesis has been studied previously^16^,^17^,^18^. However, osteogenesis remained intact despite systematic depletion of Taz in mice^19^,^20^,^21^ though a similar depletion of Taz abolished bone formation in Zebrafish^18^. Meanwhile, systematic knockout of Yap in mice caused embryonic lethality due to the failure of vasculogenesis^22^. Although most studies have shown the requirement of Yap/Taz in mesenchymal stem cells and osteoblast cells during osteogenesis *in vitro*, the role of Yap/Taz *in vivo* osteogenesis is unclear.

In this paper, we demonstrate a novel role for endothelial Yap/Taz during intramembranous ossification. Yap/Taz transcriptional activity in endothelial cells promotes intramembranous ossification via Runx2 expression. We also identified BMP4 as a bridging factor between endothelial cells and osteoblast cells. These results highlight the intertissue pathway during osteogenesis.

## Results

### Inhibition of Yap/Taz transcriptional activity in endothelial cells downregulates intramembranous ossification

**Figure 1:**
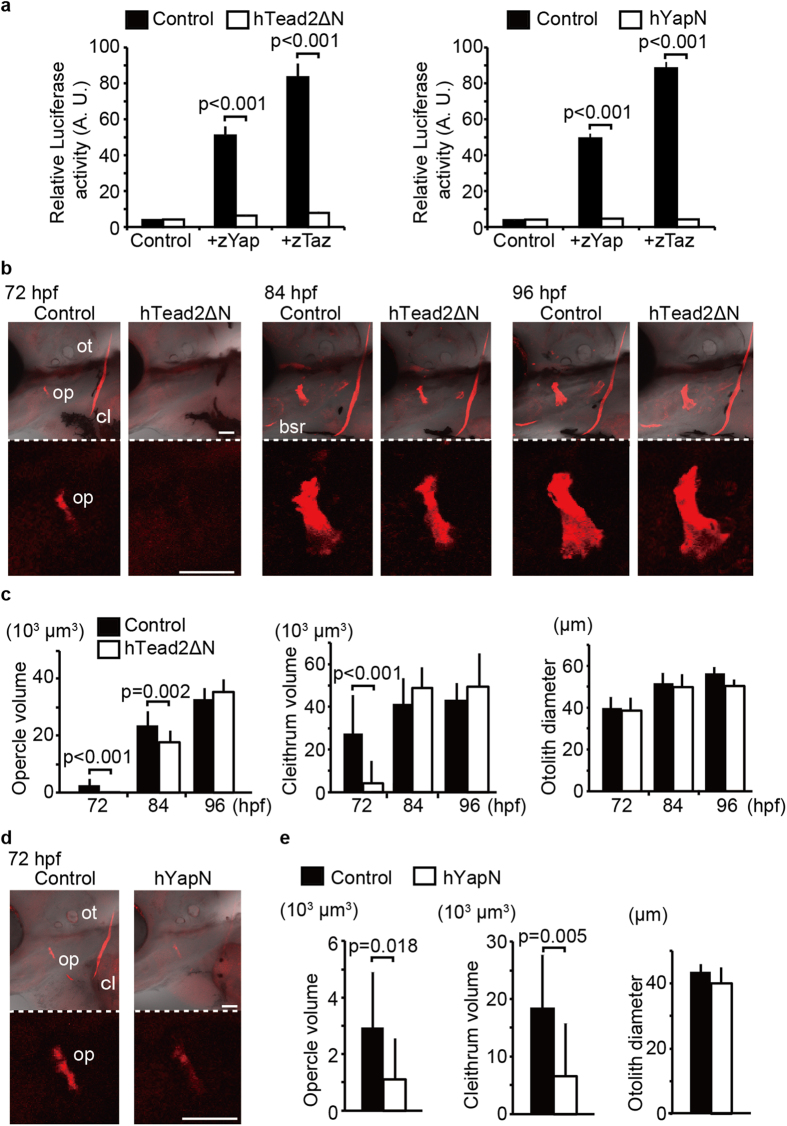
Inhibition of Yap/Taz transcriptional activity in endothelial cells downregulates intramembranous ossification. (**a**) 293T cells were transfected with pFR-Luc and pcDNA3.1-Gal4-hTead2ΔN for measuring the transcriptional activities of zYap or zTaz. Cells were also introduced with p3xflag-cmv-14-zYap, -zTaz, and pEGFP-hTead2ΔN, as indicated. 24 hours after transfection, cells were harvested and measured the transcriptional activities of zYap or zTaz by luciferase activity. Control cells were introduced pFR-Luc and pcDNA3.1-Gal4-hTead2ΔN with or without pEGFP-hTead2ΔN. Average and SD were calculated from 3 experiments. (**b**,**c**) Control *Tg*(*fli1: gal4-vp16*) embryos and *Tg*(*fli1: gal4-vp16*): (*UAS: GFP-htead2*Δ*N*) were stained with alizarin red s, and the volume of the opercle (op), cleithrum (cl), and the diameter of the otolith (ot) at indicated hours post fertilization (hpf) were measured. The Branchiostegal ray (bsr) is also indicated. Average and SD were calculated from more than 13 embryos in each sample. Bar: 50 μm. (**d**,**e**) Similar experiments to (**b**,**c**) were performed using *Tg*(*fli1: gal4-vp16*): (*UAS: GFP-hyapN*). Average and SD were calculated from more than 11 embryos in each sample. Bar: 50 μm.

Because of the location of vessels and intramembranous bones, we hypothesized that Yap/Taz transcriptional activity in endothelial cells regulates intramembranous ossification. To confirm our model, we generated two different dominant negative mutants, hTead2ΔN (amino acids 159–450) and hYapN (amino acids 47–154). The hTead2ΔN contains only C-terminal, Tead binding domain for Yap/Taz, causing endogenous Yap/Taz to fail to bind to endogenous Tead for transactivation. The hYapN consists of only the Tead binding domain and not the transactivation domain with endogenous Yap/Taz, failing to bind to authentic Tead for transactivation. To confirm that the truncated hTead2ΔN or hYapN inhibit Yap/Taz transcriptional activity, we measured the transcriptional activity of Yap/Taz based on the Gal4/UAS system (Fig. 1a and Supplemental Fig. S1)^23^. 293T cells were transfected with pFR-Luc and pcDNA3.1-Gal4-hTead2ΔN for measuring the transcriptional activities of zYap or zTaz cells. Compared with control cells introduced pFR-Luc and pcDNA3.1-Gal4-hTead2ΔN, overexpression of zYap or zTaz dramatically enhanced the luciferase activity (lane 3 and 5 in each panel), representing that Gal4-hTead2ΔN/UAS can represents the transcriptional activity of zebrafish Yap/Taz with luciferase activity. Based on these results, we also tested whether overexpression of GFP-hTead2ΔN and GFP-hYapN can inhibit Yap/Taz transcriptional activity (lane 4 and 6 in each panel). As expected, enhancements by zYap/zTaz were completely canceled by co-expressing hTead2ΔN or hYapN, representing that excess expression of hTead2ΔN or hYapN work as dominant negative mutants for zYap/zTaz transcriptional activity.

Next, we reduced Yap/Taz transcriptional activity in endothelial cells by specifically overexpressing hTead2ΔN (Fig. 1b). In this experiments, we prepared two transgenic fishlines, *Tg*(*fli1: gal4-vp16*) and *Tg*(*UAS: GFP-htead2*Δ*N*). By crossing these two lines, we obtained the fishline overexpressing GFP-hTead2ΔN in endothelial cells specifically. Intramembranous ossification was retarded in the opercle and cleithrum in embryos expressing hTead2ΔN compared to control *Tg*(*fli1: gal4-vp16*) (Fig. 1c). Meanwhile, the diameter of otoliths was little affected by overexpression of hTead2ΔN (Fig. 1c, right panel). Notably, angiogenesis in the head, especially in the opercular artery (ORA), was also little affected by expression of hTead2ΔN (Supplementary Fig. S2a). Collectively, these results suggest that Yap/Taz transcriptional activity in endothelial cells promotes early intramembranous ossification.

To confirm above results, we performed similar experiments by using *Tg*(*flk1: gal4-vp16*), using other endothelial specific promoter (Supplementary Fig. S3). The *Tg*(*flk1: gal4-vp16*): (*UAS: GFP-htead2*Δ*N*) also showed osteogenesis retardation, indicating that endothelial Yap/Taz transcriptional activity affects intramembranous ossification. We also confirmed the specificity of *Tg*(*fli1: gal4-vp16*) and *Tg*(*flk1: gal4-vp16*) by crossing *Tg*(*UAS: GFP*) whether all of endothelial cells express GFP (Supplementary Fig. S4).

We further investigated the role of Yap/Taz transcriptional activity during intramembranous ossification by overexpressing hYapN. Embryos of *Tg*(*fli1: gal4-vp16*): (*UAS: GFP-hYapN*) expressing hYapN in endothelial cells also showed similar results as Fig. 1b,c (Fig. 1d,e and Supplementary Fig. S2b). These results support our hypothesis that the inhibition of Yap/Taz transcriptional activity in endothelial cells downregulates early intramembranous ossification.

Next, we tried to identify the role of Yap/Taz transcriptional activity in osteoblast cells. To address and visualize the Yap/Taz transcriptional activity in osteoblast cells, we generated *Tg*(*osterix: gal4-htead2*Δ*N*): (*UAS: GFP*) (Supplementary Fig. S5a). Using this fish line, we observed GFP expression in the opercle and cleithrum at 99 hour postfertilization (hpf), implying that Yap/Taz was transactivated in osteoblast cells. However, the volume of the opercle in *Tg*(*osterix: gal4-vp16*): (*UAS: GFP-htead2*Δ*N*), which suppresses Yap/Taz transcriptional activity in osteoblast cells, showed subtle differences from control embryos of *Tg*(*osterix: gal4-vp16*) (Supplementary Fig. S5b,c). The specificity and expression pattern of our *Tg*(*osterix: gal4-vp16*) were confirmed by crossing *Tg*(*UAS: GFP*) (Supplementary Fig. S5d). Furthermore, we tested the volume of the opercle in *Tg*(*osterix: gal4-vp16*): (*UAS: GFP-hyap*) and *Tg*(*osterix: gal4-vp16*): (*UAS: GFP-htaz*) fish. Opercle formation in both transgenic fishes was similar to the opercle in control embryos of *Tg*(*osterix: gal4-vp16*) (data not shown). Thus, the role of Yap/Taz in osteoblasts remains uncovered.

We next investigated the role of Yap/Taz transcriptional activity in pre-osteoblast cells. To address this question, we generated *Tg*(*runx2enhancer: gal4-vp16*). Although *Tg*(*runx2enhancer: gal4-vp16*): (*UAS: GFP*) showed strong GFP signal overall in mesenchymal stem cell as reported^24^, they also showed weak GFP signals in endothelial (Supplementary Fig. S5e,f). Thus, we concluded that *Tg*(*runx2enhancer: gal4-vp16*) represents the “enhancer” activity but does not adequately represent *runx2* expression because of missing suppressors or additional regulatory factors. We also tested opercle formation using *Tg*(*runx2enhancer: gal4-vp16*): (*UAS: GFP-htead2*Δ*N*) to investigate the role of Yap/Taz transcriptional activity in pre-osteoblast cells (Supplementary Fig. S5g,h). Although opercle formation in *Tg*(*runx2enhancer: gal4-vp16*): (*UAS: GFP-htead2*Δ*N*) was moderately retarded, the possibility of an importance of endothelial Yap/Taz remains because GFP was expressed in endothelial cells in *Tg*(*runx2enhancer: gal4-vp16*): (*UAS: GFP*).

### Overexpression of Yap/Taz in endothelial cells promotes intramembranous ossification

**Figure 2:**
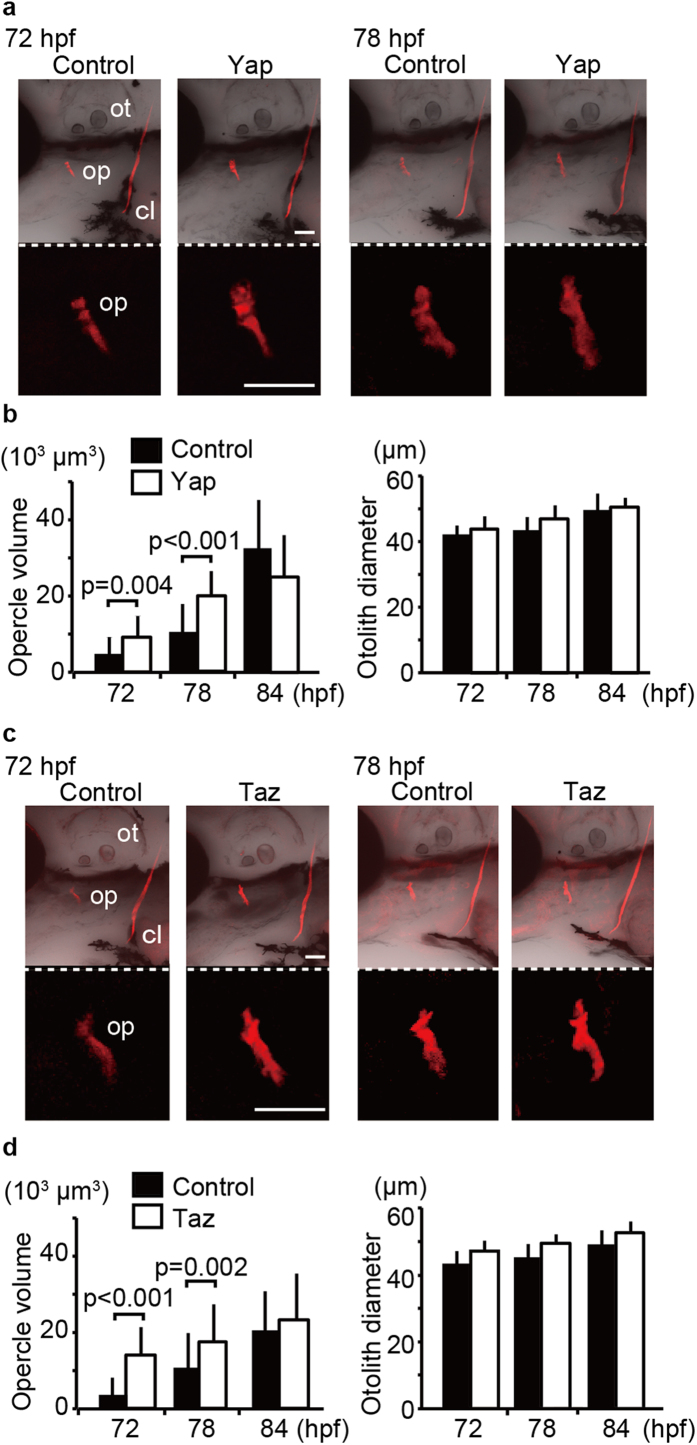
Overexpression of Yap/Taz in endothelial cells promotes intramembranous ossification. (**a**) Control *Tg*(*fli1: gal4-vp16*) embryos and *Tg*(*fli1: gal4-vp16*): (*UAS: GFP-hyap*) were stained with alizarin red s, and the volume of the opercle (op) was measured at the indicated hpf. Average and SD were calculated from more than 23 embryos in each sample. Bar: 50 μm. (**b**) Similar experiments as (**a**) were performed with *Tg*(*fli1: gal4-vp16*): (*UAS: GFP-htaz*). Average and SD were calculated from more than 35 embryos in each sample. Bar: 50 μm.

To confirm that endothelial Yap/Taz is responsible for intramembranous ossification, we tested whether forced expression of Yap/Taz in endothelial cells affects early intramembranous ossification, because dominant-negatives could also affect pathways other than Yap/Taz transactivation. In these experiments, we focused on the size of the opercle, because it is one of the earliest intramembranous ossifications in zebrafish (Fig. 2). Embryos overexpressing Yap and Taz in endothelial cells showed larger opercle volumes in very early ossification compared to controls. Notably, cartilaginous tissues in head were little affected by expression of hTead2ΔN or Yap in endothelial cells (Supplementary Fig. S6a). Thus, we concluded that Yap/Taz transcriptional activity in endothelial cells promotes intramembranous ossification.

### Blood flow is required for Yap/Taz transcriptional activation in ORA and intramembranous ossification

**Figure 3:**
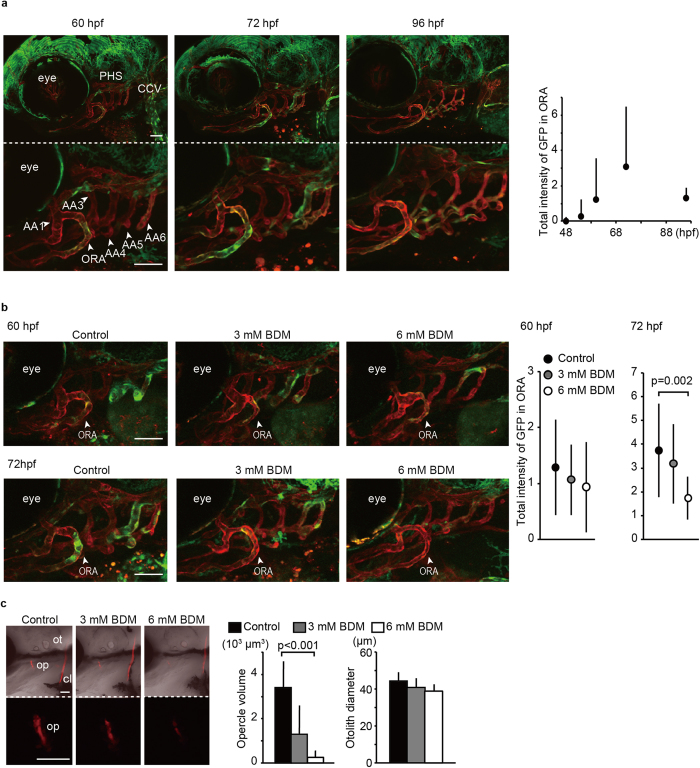
Blood flow is required for Yap/Taz transcriptional activation in ORA and intramembranous ossification. (**a**) Stacked images of *Tg*(*fli1: gal4-htead2*Δ*N*): (*UAS: GFP*): (*fli1: myr-mCherry*) at the indicated hpf are shown. Green color represents Yap/Taz transcriptional activity. Red color represents all endothelial cells. The opercular artery (ORA), primary head sinus (PHS), common cardinal vein (CCV), and branchial arteries (AA) are also indicated. Average and SD of the total GFP intensity in ORA were measured from 20 embryos at each time-point and are shown in the right panel. Bar: 50 μm. (**b**) Similar experiments to (**a**) were performed with BDM treatment. Embryos without BDM treatment also shows as control. *Tg*(*fli1: gal4-htead2*Δ*N*): (*UAS: GFP*): (*fli1: myr-mCherry*) were treated with BDM from 54 hpf to the indicated hpf. Average and SD of the total GFP intensity in the ORA were measured from more than 18 embryos at each time-point. Bar: 50 μm. (**c**) Embryos were treated with or without BDM from 54 hpf to 72 hpf, stained with alizarin red s, and the volume of the opercle (op) and the diameter of the otolith (ot) were measured. Embryos without BDM treatment also shows as control. Average and SD were calculated from more than 10 embryos in each sample. Bar: 50 μm.

Next we endeavored to verify whether Yap/Taz transcriptional activity does indeed occur in endothelial cells during opercle formation. For this purpose, we generated a fish line for monitoring Yap/Taz transcriptional activity specifically in endothelial cells. The fish line expresses Gal4-hTead2ΔN under the control of the fli1 promoter. We crossed this line with *Tg*(*UAS: GFP*): (*fli1: myr-mCherry*) to visualize the activity of Yap/Taz only in endothelial cells. Since endogenous Yap/Taz can bind to hTead2ΔN, Yap/Taz is recruited on GFP coding region via Gal4/UAS binding. These *Tg*(*fli1: gal4-htead2*Δ*N*): (*UAS: GFP*): (*fli1: myr-mCherry*) exhibited GFP signals in the opercular artery (ORA) during opercle development between 48 to 96 hpf (Fig. 3a), indicating that Yap/Taz transcriptional activity increased in the ORA during opercle generation.

Since Yap/Taz transcriptional activity is regulated by mechanical stress factors, we hypothesized that shear stress derived from circulation regulates Yap/Taz transcriptional activity in the ORA (Fig. 3b). We treated embryos with 2, 3-Butanedione monoxime (BDM), which is an inhibitor of myosin ATPase and suppresses cardiac contraction to decrease circulation volume and blood pressure. Embryos treated with 3 mM and 6 mM BDM from 54 hpf to 60 hpf and to 72 hpf showed less GFP signal in the ORA, indicating that Yap/Taz transcriptional activity in the ORA is upregulated by blood flow. Furthermore, in similar conditions, opercle formation was abrogated by BDM treatment (Fig. 3c), while cartilaginous tissues in head were little affected (Supplementary Fig. S6b). Thus, we concluded that blood flow is required for Yap/Taz transcriptional activation in the ORA and intramembranous ossification.

### Yap/Taz transcriptional activation in endothelial cells promotes *runx2* expression

**Figure 4:**
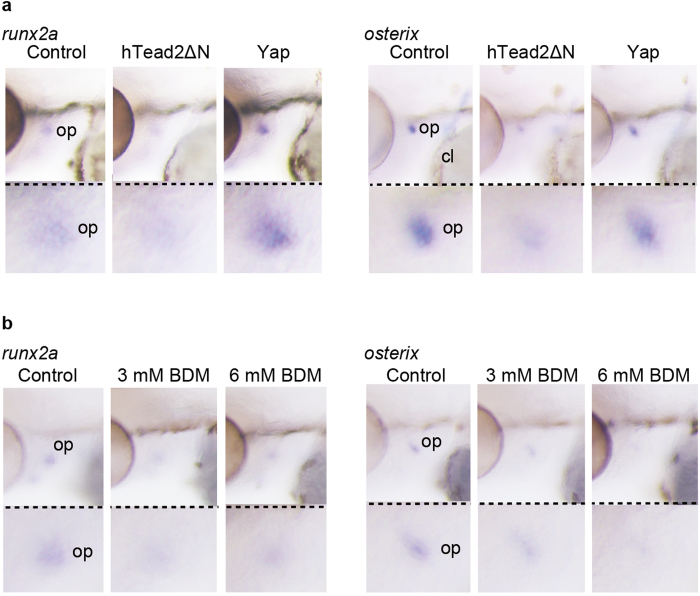
Yap/Taz transcriptional activation in endothelial cells promotes *runx2* expression. (**a**) Control *Tg*(*fli1: gal4-vp16*), *Tg*(*fli1: gal4-vp16*): (*UAS: GFP-htead2*Δ*N*), and *Tg*(*fli1: gal4-vp16*): (*UAS: GFP-hyap*) were fixed at 60 hpf with paraformaldehyde, and WISH was performed to detect the mRNA of *runx2a* and *osterix*. (**b**) Similar experiments to (**a**) were performed. Embryos of *Tg*(*fli1: gal4-vp16*), were treated with BDM from 54 to 72 hpf at the indicated dosage.

Since our results demonstrated that endothelial Yap/Taz regulates early intramembranous ossification, we hypothesized that Yap/Taz affects differentiation during osteogenesis. To confirm this model, we tested the expression levels of *runx2* and *osterix* as markers for pre-osteoblasts and osteoblasts, respectively (Fig. 4a). To manipulate Yap/Taz transcriptional activity in endothelial cells, we prepared embryos expressing hTead2ΔN or Yap in endothelial cells at 60 hpf and performed whole mount *in situ* hybridization (WISH). Compared to the controls, hTead2ΔN-expressing fish showed less *runx2* signals. Furthermore, Yap-overexpressing fish showed stronger *runx2* signals, indicating that Yap/Taz transcriptional activation in endothelial cells promotes *runx2* expression. In the case of *osterix*, the signal in Yap-overexpressing fish was comparable to the control, though hTead2ΔN-expressing fish showed less *osterix* signal. These data support the hypothesis that *runx2* is essential for *osterix* expression but not sufficient. In summary, Yap/Taz transcriptional activation in endothelial cells promotes *runx2* expression specifically during intramembranous ossification. We also performed experiments with BDM, which suppresses Yap/Taz transcriptional activity in ORA, similar to those shown in Fig. 3. Treatment with BDM from 54 to 60 hpf reduced the expression levels of *runx2* and *osterix* in a dose dependent manner (Fig. 4b), supporting our model that Yap/Taz transcriptional activation in endothelial cells is required for *runx2* expression in pre-osteoblast cells.

### BMP pathway upregulates *runx2* expression during opercle formation

**Figure 5:**
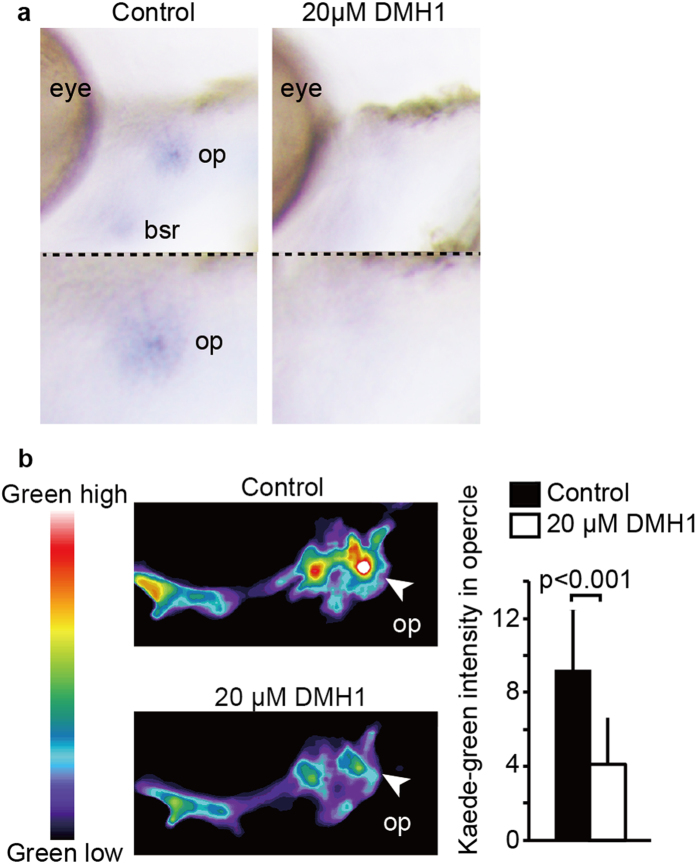
BMP pathway upregulates *runx2* expression during opercle formation. (**a**) Embryos of *Tg*(*fli1: gal4-vp16*) were treated with or without 20 μM DMH1 from 54 to 72 hpf, fixed, and WISH was performed to detect the mRNA of *runx2a*. (**b**) *Tg* (*runx2enhancer: gal4-vp16*): (*UAS: kaede*) were treated with or without DMH1 from 54 hpf, irradiated with UV at 60 hpf, and Kaede-green intensity in the opercle area was measured at 72 hpf. Representative images are shown with pseudo color. Average and SD of Kaede-green intensity were calculated from more than 22 embryos in each sample, and are shown in right panel.

We next investigated the bridging molecules/pathway between blood vessels and intramembranous ossification. Given the above results, we hypothesized that a secreted downstream factor of Yap/Taz might promote osteogenesis. The bone morphogenetic protein (BMP) is a family of secreted proteins that are well known osteogenic factors and promote runx2 expression^25^. Hence, we tested whether the BMP pathway regulates *runx2* expression in the opercle by using DMH1, which is an inhibitor of the BMP receptor (Fig. 5a). Compared to the controls, DMH1 treatment significantly reduced the expression level of *runx2*, showing that the BMP pathway is essential for *runx2* expression. To confirm the inhibitory effect of DMH1 with a complimentary approach, we generated *Tg* (*runx2enhancer: gal4-vp16*): (*UAS: kaede*) to monitor the enhancer activity of *runx2*. Kaede is a fluorescence protein, which changes color from green to red by photoconversion under ultra violet (UV) radiation. Using this property of Kaede, we were able to improve the temporal resolution in our monitored fish. Embryos were treated with DMH1 from 54 hpf and irradiated with UV at 60 hpf, and then Kaede-green intensity was measured at 72 hpf in the opercle area (Fig. 5b). Compared to the controls, DMH1 treatment significantly reduced Kaede-green intensity. These results further support our hypothesis that the BMP pathway is essential for *runx2* expression in opercle development.

### Yap/Taz transcriptional activity is required for *bmp* expression in endothelial cells

**Figure 6:**
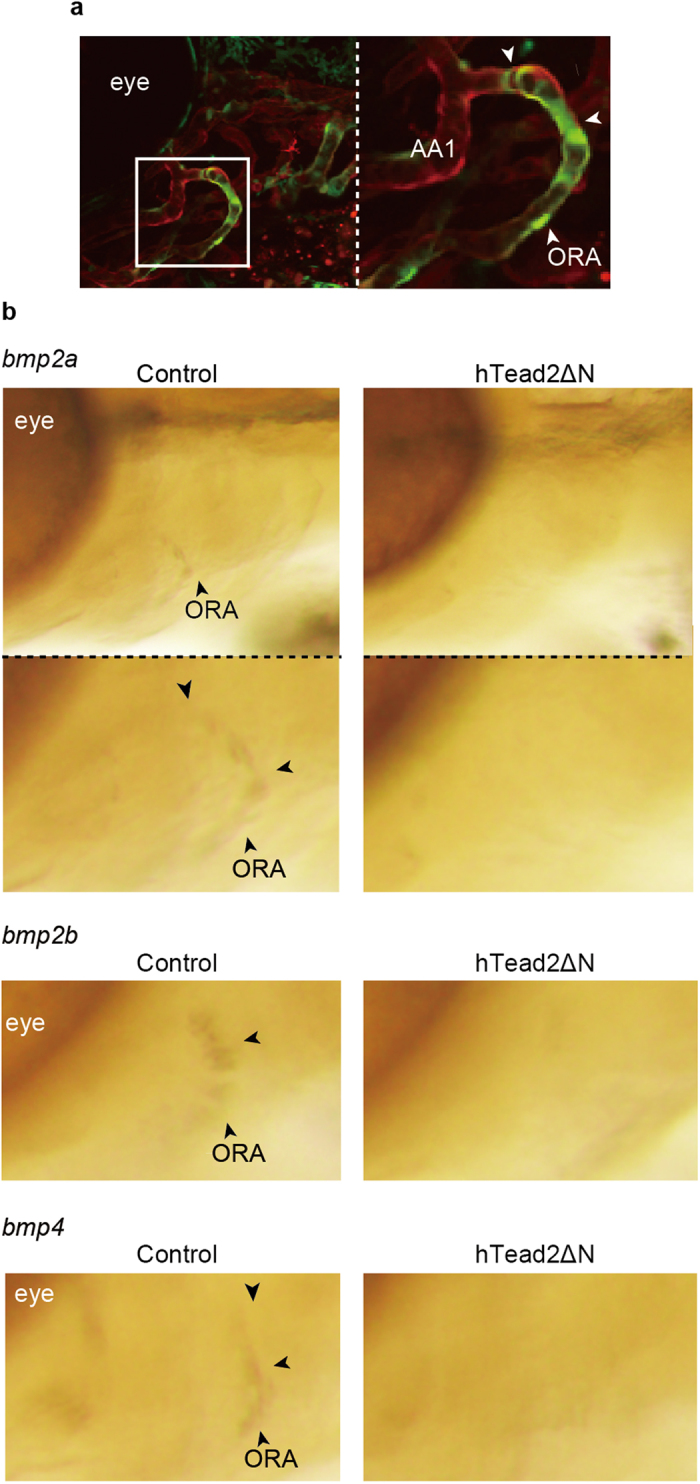
Yap/Taz transcriptional activity is required for bmp expression in ORA. (**a**) Representative images of ORA in *Tg*(*fli1: gal4-htead2*Δ*N*): (*UAS: GFP*): (*fli1: myr-mCherry*) at 72 hpf are shown. Green color represents Yap/Taz transcriptional activity. Red color represents all endothelial cells. (**b**) Control *Tg*(*fli1: gal4-vp16*) and embryos of *Tg*(*fli1: gal4-vp16*): (*UAS: GFP-htead2*Δ*N*) were fixed with glutaraldehyde at 72 hpf, and WISH was performed to detect mRNA of *bmp2a*, *2b*, and *4*.

We next tested whether the BMP family is indeed expressed in the ORA via Yap/Taz transcriptional activity. Since Bmp2a, 2b, and 4 have already been reported as secreted proteins from blood vessels, we focused on these homologs. In these experiments, we used glutaraldehyde for fixation because vascular structure in embryos fixed with paraformaldehyde collapsed during WISH experiments (Fig. 6a,b). At 72 hpf, signals from *bmp2a*, *2b*, and *4* were detected specifically in the ORA using control embryos, while these signals were diminished in embryos expressing hTead2ΔN in endothelial cells. Notably, there were no remarkable signals in the opercle, suggesting that *bmp* expression is not derived from the opercle itself. Thus, our results demonstrate that Yap/Taz transcriptional activation in the ORA promotes *bmp* expression from endothelial cells during intramembranous ossification.

### Bmp4 expression from endothelial cells promotes opercle formation

**Figure 7:**
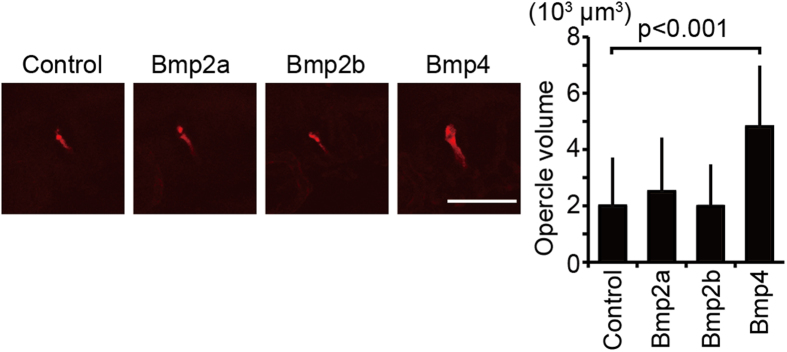
Bmp4 is a target of Yap/Taz during opercle formation. Plasmids containing *UAS: bmp2a*, *UAS: bmp2b*, and *UAS: bmp4* were introduced to embryos of *Tg*(*fli1: gal4-vp16*) at the one-cell stage. Un-injected embryos were prepared as control. Embryos were stained with alizarin red s, and the volume of the opercle (op) at 72 hpf was measured. Average and SD were calculated from more than 18 embryos in each sample. Bar: 50 μm.

Finally, we investigated which Bmp from endothelial cells is responsible for opercle formation. We prepared embryos overexpressing Bmp2a, 2b, and 4 in endothelial cells, and measured the volume of opercles at 72 hpf (Fig. 7). Compared to controls, Bmp4 expression in endothelial cells clearly enlarged the opercle, however the overexpression of Bmp2a and 2b did not. Thus, we concluded that Bmp4 is a bridging molecule between blood vessels and intramembranous ossification.

To determine that endothelial Yap/Taz-Bmp4 pathway is a sufficient factor in intramembranous ossification, we tested whether the effect of endothelial GFP-htead2ΔN could be cancelled by Bmp4 overexpression (data not shown). The Bmp4 overexpression in *Tg*(*fli1: gal4-vp16*): (*UAS: GFP-htead2*Δ*N*) still showed smaller opercles, implying that Bmp4 is not only a target of endothelial Yap/Taz in osteogenesis.

## Discussion

In this paper, we demonstrated the intertissue relationship between blood flow and osteogenesis in zebrafish. Circulation activates Yap/Taz transcriptional activity in endothelial cells and transactivated Yap/Taz promotes Bmp4 expression in endothelial cells, and Runx2 expression in precursor cells of the opercle for its ossification.

The relationship between blood vessels and intramembranous ossification is poorly understood^2^. Due to the location of vessels and intramembranous bones, the linkage between these two has been implicated that vessels regulates intramembranous bone generations^26^. A previous genetic study also showed the importance of blood vessels for intramembranous ossification^6^. Thus, vascular structure has been regarded as a regulator for intramembranous ossification. However, though most studies have shown defects in intramembranous ossification related to undeveloped vascular structure, the molecular linkages between blood vessels and intramembranous ossification remained unidentified. Our results demonstrate, for the first time, that endothelial Yap/Taz is a key factor in intramembranous ossification but has little effect on angiogenesis, at least in the opercular artery.

In our study, manipulation of Yap/Taz transactivation in endothelial cells affected the very early phase of intramembranous ossification but the effect was negated in later stages. We hypothesize that Yap/Taz transactivation has two roles, promoting early intramembranous ossification and inhibiting bone maturation in later phase. Indeed, GFP expression of *Tg*(*fli1: gal4-htead2*Δ*N*): (*UAS: GFP*) in the ORA was transient. These results imply that Yap/Taz transactivation may be tightly regulated during fish development, and should be downregulated at certain stages. The expression of Runx2 as a target of Yap/Taz transactivation may explain the bipolar function of Yap/Taz transactivation in endothelial cells during intramembranous ossification. Runx2 has been shown to be a master regulator of osteogenesis including intramembranous ossification^27^,^28^, while forced expression of Runx2 in osteoblast cells results in defective osteogenesis presenting as osteopenia^29^. Our results thus suggest another possible mechanism governing the interaction between endothelial cells and osteoblast cells based on Yap/Taz regulation.

The role of Yap/Taz transactivation in osteoblast cells has remained undefined. Although several studies showed the requirement of Yap/Taz in osteogenesis^17^,^18^,^30^, most in *in vivo* studies in mice, by depleting Taz showed subtle effects on osteogenesis^19^,^20^,^21^,^22^. Here, we succeeded in detecting the transactivation of Yap/Taz in osteoblast cells during intramembranous ossification. Meanwhile, the size of the opercle was similar to the controls in early intramembranous osteogenesis by upregulation and downregulation of Yap/Taz transactivation in osteoblast cells. It is possible that Yap/Taz transactivation is required in a later phase of homeostasis of bone formation. Further studies are required to understand the role of Yap/Taz transactivation in osteoblasts.

We hypothesized that Yap/Taz transactivation in endothelial cells may be also involved in pathological ossification, such as arteriosclerosis. Since lesions of arteriosclerosis are frequently found near the branching point in the aorta where turbulent flow occures^31^,^32^,^33^, several studies have implied that turbulent flow is a factor in arteriosclerosis. Additionally, recent studies have shown that Yap/Taz transactivation was dramatically enhanced by mechanical stress including flow^15^,^34^,^35^. Notably, our results show that endothelial BMP4 and osteoblast Runx2 are bridging factors between blood vessels and osteogenesis. Similar to our model, BMP in endothelial cells^36^,^37^ and Runx2 in vascular smooth muscle^38^,^39^ are also key factors for arteriosclerosis in mice. In summary, our results provide clues towards understanding the relationship between turbulent flow and ectopic ossification.

## Materials and Methods

### Zebrafish husbandry

Zebrafish (*Danio rerio*) strains were maintained under standard conditions. We used a fish medium containing 0.03% sea salt and 0.006% methylene blue as an antiseptic agent. Embryo stages were determined from hpf at 28 °C^40^. Microinjections and chemical treatment of embryos were undertaken as described below. All experiments using zebrafish were carried out with approval from the institutional ethics committee of the University of Tokyo, Japan, strictly following the guidelines set for the usage of animals by this committee.

### Plasmid constructs

To prepare plasmids for generating zebrafish lines, we constructed *pTol2-fli1-gal4-htead2*Δ*N-2A-mCherry*, *pTol2-UAS-GFP-htead2*Δ*N*, *pTol2-UAS-GFP-hyapN*, *pTol2-UAS-GFP-hyap*, *pTol2-UAS-GFP-htaz*, *pTol2-runx2enhancer-gal4-vp16-2A-mCherry*, *pTol2-runx2enhancer-gal4-htead2*Δ*N-2A-mCherry*, *pTol2-osterix-gal4-vp16-2A-mCherry*, and *pTol2- osterix-gal4-htead2*Δ*N-2A-mCherry*. The Tol2 vector system was kindly gifted by K. Kawakami (National Institute of Genetics, Japan)^41^. M. Hibi (Nagoya University, Japan) provided the UAS sequence. cDNA fragments encoding human Tead2, Yap, and Taz were amplified by PCR from cDNA libraries. The Runx2 enhancer and Osterix promoter were amplified and subcloned as described previously^24^,^42^. The genomic library of Medaka was kindly gifted by H. Nishina (Tokyo Medical and Dental University, Japan). cDNA for expressing and detecting zYap, zTaz, Runx2a, Osterix, Bmp2a, Bmp2b, and Bmp4 were also amplified by PCR from cDNA libraries of zebrafish.

### Transgenic zebrafish lines

Tol2 transposase mRNA was synthesized *in vitro* with SP6 RNA polymerase from a NotI linearized pCS-TP vector. To generate transgenic zebrafish lines, the corresponding Tol2-based DNAs (100 ng/μl) were microinjected along with Tol2 transposase mRNA (25 ng/μl) into one-cell stage embryos of the wild type strain AB. We generated *Tg*(*fli1: gal4-htead2*Δ*N-2A-mCherry*), *Tg*(*UAS: GFP-htead2*Δ*N*), *Tg*(*UAS: GFP-hyapN*), *Tg*(*UAS: GFP-hyap*), *Tg*(*UAS: GFP-htaz*), *Tg*(*runx2enhancer: gal4-vp16-2A-mCherry*), *Tg*(*osterix: gal4-vp16-2A-mCherry*), and *Tg*(*osterix: gal4-htead2*Δ*N-2A-mCherry*).

The *Tg*(*UAS: GFP*) fish line was kindly provided by M. Hibi^43^, and the *Tg*(*fli1: gal4-vp16*) fish line was a gift from M. Affolter (University of Basel, Switzerland)^44^. The *Tg*(*flk1: gal4-vp16*)*, Tg*(*fli1: Myr-mCherry*) and *Tg*(*UAS: mCherry*) fish lines were provided by N. Mochizuki^45^,^46^. The *Tg*(*UAS: Kaede*) fish line was kindly provided by Baier H. (Max Planck Institute of Neurobiology, Munich)^47^.

### Image acquisition, processing, and quantification

Zebrafish embryos were dechorionated and mounted in 1% low-melting agarose on a 35 mm glass bottomed dish (Asahi Techno Glass) with 0.016% tricaine (Sigma-Aldrich) in fish medium as described previously^48^. The dish was submerged in fish medium containing 0.001% tricaine. Bone staining with alizarin red s (Wako) was performed as per the manufacturer’s protocol.

Confocal images were obtained with an FV1000 confocal upright microscope system (Olympus) equipped with a 20× water-immersion lens (XLUMPlanFL, NA 1.0). 473 nm and 559 nm laser lines were employed for green fluorescence protein (GFP and Kaede-green), and red fluorescence molecules (mCherry and alizarin red s), respectively. Image files were processed and analyzed using FLUOVIEW Viewer software (Olympus), MetaMorph (Molecular Devices), and Volocity (perkinelmer).

### Chemical treatment of zebrafish embryos

Zebrafish embryos were treated with BDM and DMH1 (Sigma-Aldrich) in fish medium.

### Whole-mount *in situ* hybridization

Whole-mount *in situ* hybridization of zebrafish embryos was performed as described previously^49^. Pigmentation of embryos was inhibited by 0.04 mM 1-phenyl-2-thiourea (PTU) (Sigma-Aldrich) from 8 hpf onwards.

### Microinjection of plasmid to zebrafish embryos

Plasmid DNA (100 ng/μl) mixed with injection buffer (120 mM KCl, 20 mM Hepes, 0.25% phenol red) was microinjected into embryos in their one-cell stage.

### Statistical analysis

Data are expressed as the average ± SD. The statistical significance for paired samples was determined using Student’s t-test.

## Additional Information

**How to cite this article**: Uemura, M. *et al*. Yap/Taz transcriptional activity in endothelial cells promotes intramembranous ossification via the BMP pathway. *Sci. Rep*. **6**, 27473; doi: 10.1038/srep27473 (2016).

## Supplementary Material

Supplementary Information
