# Supplementary material for: Yap/Taz transcriptional activity in endothelial cells promotes intramembranous ossification via the BMP pathway
Source: Sci Rep. 2016 Jun 7;6:27473. doi: 10.1038/srep27473 (PMC4895351; doi:10.1038/srep27473)
Supplement: Supplementary Information [file srep27473-s1.pdf]

Supplementary Information

Yap/Taz transcriptional activity in endothelial cells promotes intramembranous ossification via the BMP pathway

Mami Uemura[a], Ayumi Nagasawa[a], and Kenta Terai[a*]

[a]Laboratory of Function and Morphology,

Institute of Molecular and Cellular Biosciences,

The University of Tokyo.

Yayoi 1-1-1 Bunkyo-ku Tokyo 113-0032 Japan

Phone: +81-3-5841-7836

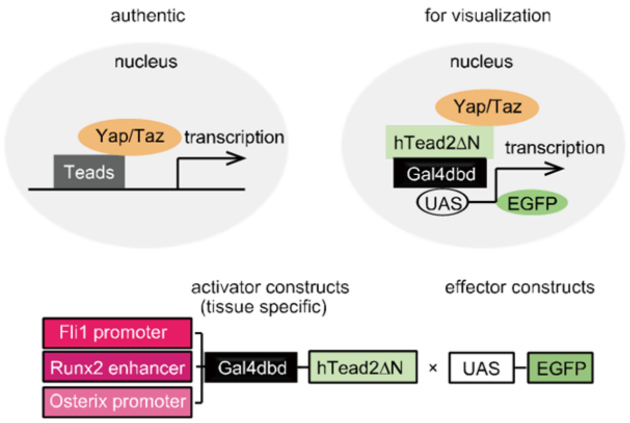

Supplemental Fig. S1. Shema represents how visualize Yap/Taz transcriptional activity in specific cells.

In authentic cells, endogenous Yap and/or Taz translocate into nuclear via Teads binding. Based on such a model, we introduced Gal4-hTead2ΔN derived transcribed by tissue specific promoter such as Fli1, Runx2, or Osterix. Since fish contains GFP coding sequence under UAS, we can visualize Yap/Taz transcriptional activity via GFP signal.

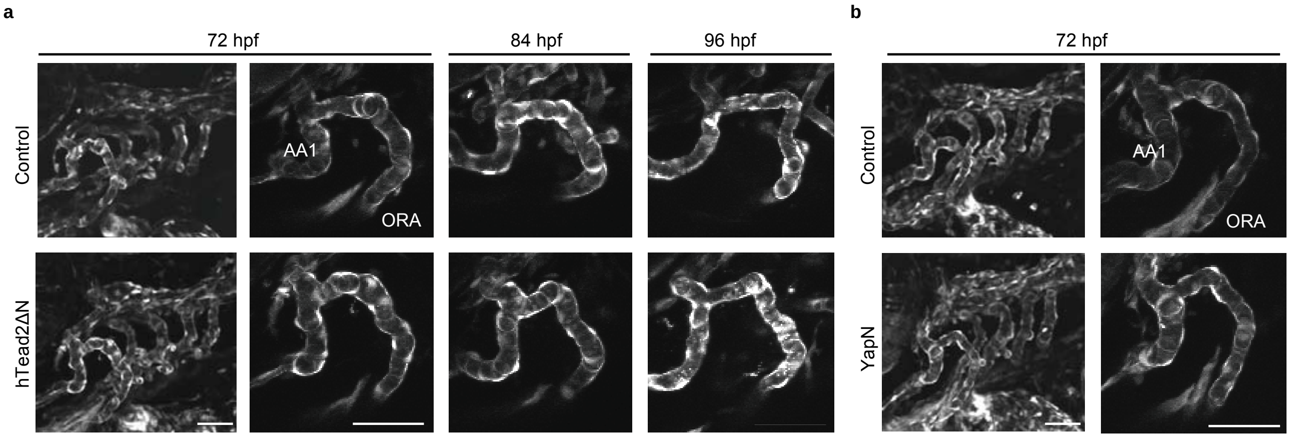

Supplemental Fig. S2. Inhibition of Yap/Taz transcriptional activity in endothelial cells affects little in head angiogenesis, especially in the ORA.

(**a**) and (**b**) Control embryos, *Tg(fli1: gal4-vp16): (UAS: GFP-htead2ΔN)*, and *Tg(fli1: gal4-vp16): (UAS: GFP-hyapN)* were crossed with *(UAS: mCherry)*. mCherry expression was visualized in white color as endothelial marker at indicated time. Bar: 50 µm.

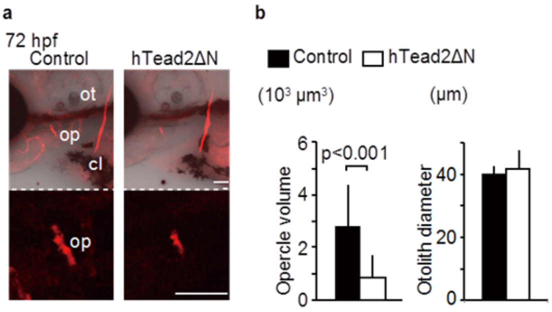

Supplemental Fig. S3. The *Tg(flk1: gal4-vp16): (UAS: GFP-htead2ΔN)* also shows osteogenesis retardation.

(**a**) and (**b**) Control *Tg(flk1: gal4-vp16)* embryos and *Tg(flk1: gal4-vp16): (UAS: GFP-htead2ΔN)* were stained with alizarin red s, and the volume of the opercle (op) and the diameter of the otolith (ot) at indicated hours post fertilization (hpf) were measured. Average and SD were calculated from more than 15 embryos in each sample. Bar: 50 μm.

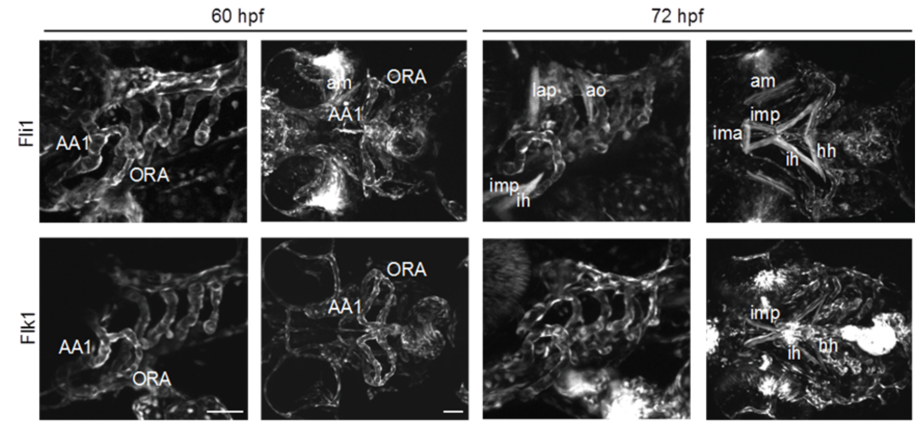

Supplemental Fig. S4. The *Tg(fli1: gal4-vp16)* and the *Tg(flk1: gal4-vp16)* selectively express *gal4-vp16* gene in endothelial cells.

The *Tg(fli1: gal4-vp16)* and the *Tg(flk1: gal4-vp16)*were crossed with *Tg(UAS: GFP)* and analyzed at indicated hpf. Branchial arteries (AA), opercular artery (ORA), adductor mandibulae (am), levatorarcus palatini (lap), adductor operculae (ao), intermandibularis posterior (imp), interhyoideus (ih), intermandibularis anterior (ima), intermandibularis posterior (imp), and hyohyoideus (hh) are also indicated. Bar: 50 µm.

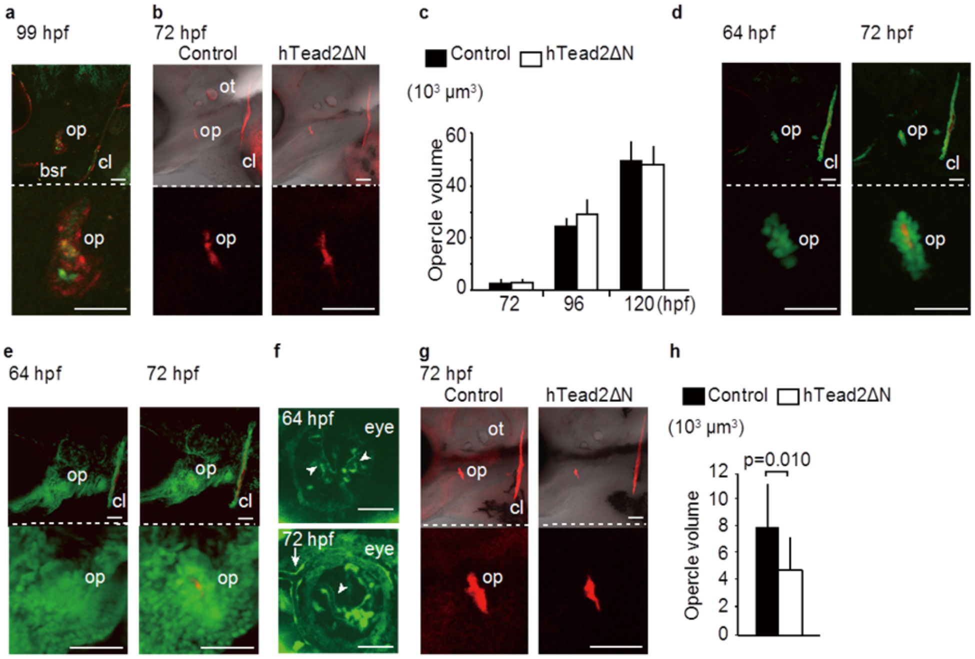

Supplemental Fig. S5. Inhibition of Yap/Taz transcriptional activity in osteoblast cells affects little in the opercle formation.

(**a**) Stacked images of *Tg(osterix: gal4-htead2ΔN): (UAS: GFP)* at indicated hpf are shown. Green color represents Yap/Taz transcriptional activity. Bar: 50 µm. (**b**) and (**c**) Control embryos of *Tg(osterix: gal4-vp16)* and *Tg(osterix: gal4-vp16): (UAS: GFP-htead2ΔN)* were stained with alizarin red s, and measured the volume of opercle (op) at indicated hpf. Average and SD are calculated with more than 5 embryos in each sample. Bar: 50 µm. (**d**) Representative images of *Tg(osterix: gal4-vp16): (UAS: GFP)* at indicated hpf are shown. Bar: 50 µm. (**e**) and (**f**) Stacked images of *Tg(runx2enhancer: gal4-vp16): (UAS: GFP)* at indicated hpf are shown. Green color represents GFP expression. In (f), a stacked image

in longer exposure is shown. In upper panel, left arrowheads indicate hyaloid vessels. In lower panel, left arrows indicates superficial annular vessel. Right arrowhead indicates hyaloid vessels. Bar: 50 µm. (**g**) and (**h**) Similar experiments were performed as (b) and (c) by using *Tg(runx2enhancer: gal4-vp16): (UAS: GFP-htead2ΔN)*. Average and SD are calculated with more than 10 embryos in each sample. Bar: 50 µm.

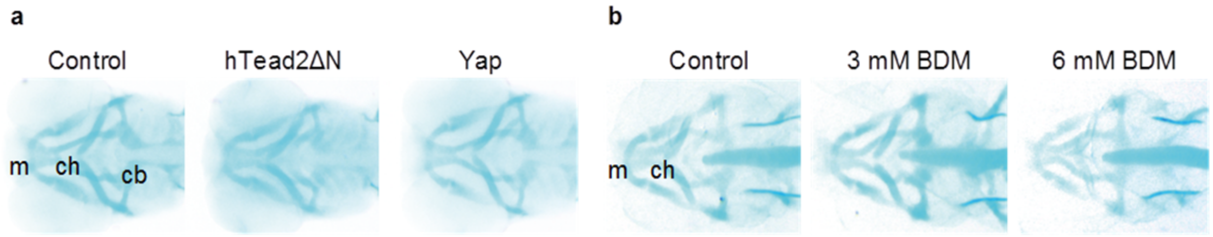

Supplemental Fig. S6. Inhibition of Yap/Taz transcriptional activity in endothelial cells or blood circulation affects little in head endochondral development.

(**a**) Control *Tg(fli1: gal4-vp16), Tg(fli1: gal4-vp16): (UAS: GFP-htead2ΔN), and Tg(fli1: gal4-vp16): (UAS: GFP-hyap)* were fixed at 72 hpf and stained with alcian blue for detecting cartridge. (**b**) Similar experiments were performed as (a). Control *Tg(fli1: gal4-vp16)* were treated with BDM from 54 hpf to 72 hpf. Meckel's (m), ceratohyal (ch), and ceratobranchial (cb) cartilage are indicated.
